# A sheeppox outbreak in Morocco: isolation and identification of virus responsible for the new clinical form of disease

**DOI:** 10.1186/1746-6148-10-31

**Published:** 2014-01-27

**Authors:** Khalil Zro, Fathiah Zakham, Marouane Melloul, Elmostafa El Fahime, Moulay Mustapha Ennaji

**Affiliations:** 1 Laboratory of Virology, Hygiene & Microbiology, Faculty of Science and Techniques, University Hassan II. Mohammedia_Casablanca, BP 146, Mohammedia 20650 Morocco; 2 Laboratoire Régional d’Analyses et de Recherches d’Oujda, Office National de Sécurité Sanitaire des Produits Alimentaires, BP 3136, Route d’Ahfir, 60000 Oujda, Morocco; 3 Plateforme Génomique Fonctionnelle UATRS-Biologie –CNRST, Rabat, Morocco

**Keywords:** Sheeppox, Real-time PCR, Phylogenetic analysis, TK gene, IL8 gene

## Abstract

**Background:**

Sheeppoxvirus (SPPV) is a member of the Capripoxvirus genus of the *Poxviridae* family, which causes significant economic losses in Morocco. The resurgence of the sheeppox disease during 2010 was characterized by an emergence of a classical nodular form for the first time in Morocco. However, little is known about the virus strain responsible for nodular form. In this study, thirty three sheep, from the eastern region of Morocco, clinically infected were examined and dead animals were autopsied.

A rapid diagnostic assay for SPPV using different type of clinical samples would be useful for outbreak management. The aim of this work was to isolate the virus strain responsible for nodular form and we identified and compared by phylogenetic analysis the field strain with Moroccan vaccine strain targeting the thymidine kinase (TK) gene and the chemokine analogue receptor of interleukin (IL8) gene. Further, it was important to investigate and validate a real-time PCR using different clinical and post-mortem samples to manage epidemic sheeppox disease.

**Results:**

The nodular form of sheeppox disease observed in Morocco was clinically characterized by fever, depression, lacrimation, diarrhea in lambs and nodule. At necropsy, the most affected organ was the lung. The etiological strain was successfully isolated from lung nodule in a dead lamb and was identified by using real-time PCR that has been tested and validated on different types of clinical and post mortem samples from naturally infected animals. Sequence and phylogenetic analysis of TK and IL8 gene showed that there was a very close relationship between field and vaccine strain. They were clustered within other SPPV strains.

**Conclusion:**

In the current study, we show for the first time the nodular form of sheeppox in Morocco. We demonstrate a robust real-time PCR-based diagnostic assay to detect the sheeppox virus in multiple sample that can be implemented to efficiently manage the disease outbreak. Our study also offers the prospect for future molecular studies to understand the clinical forms.

## Background

Sheeppox virus (SPPV) is a member of Capripoxviruses (CaPVs) genus, which belongs to the *Poxviridae* family [1]. CaPVs are generally considered to be host-specific: Lumpy Skin Disease virus (LSDV) affects cattle and is currently found in most African countries and the Middle East. In contrast, SPPV and goatpox virus (GTPV) affect both sheep and goats and occur in Africa, the Middle East and Asia [2,3].

In Morocco, for instance after decades of vaccination, SPPV is still responsible for substantial economic losses in sheep breading [4]. Sheeppox disease appears only in sheep and no case affecting goats has been reported and it seems that the circulated virus for several years in different parts of the kingdom merely affects sheep.

Further, the clinical classic vesicular form was usually observed and characterized by the appearance of skin lesions on the entire body surface evolving from macules, papules, vesicles or vesiculo- pustules and crusts at the end of disease evolution [5]. However, during the epizootic outbreaks recorded during 2010 in the Eastern region of Morocco, we exclusively noted for the first time the appearance of a new form, called the nodular form and was qualified as severe in some cases. This clinical form has been reported in Mauritania [6] and reproduced following experimental inoculation of animals with SPPV strains isolated in some countries in West Africa [7,8].

Diagnosis of sheeppox is usually based on highly characteristic clinical signs [9], virus isolation, virus neutralisation test (VNT) [10], serological assays (e.g. ELISA) [11] and PCR assays [12].

The aim of this work is to describe for the characteristics of clinical nodular form observed for the first time in Morocco and to test the real time PCR described by Balinsky using different types of samples collected from naturally infected sheep.

In this present study, the confirmation of the nodular form of SPPV was done by viral isolation and molecular investigations in an epidemiological context, where serology did not meet the need. PCR was used in blood, nasal swabs, oral swabs, rectal swabs and crusted scabs as well as in lung nodule collected at necropsy to manage outbreaks.

Furthermore, automated sequencing was performed by targeting the thymidine kinase gene (TK) and chemokine analogue receptor of interleukin gene (IL8). The results were used for phylogenetic analysis by comparing Moroccan vaccine strain with Moroccan field strain and other various Capripoxvirus (CaPV) isolates retrieved from GenBank to elucidate the genetic relatedness of these viruses.

## Methods

### Outbreaks investigation

During the first half of 2010, an epidemic of sheeppox disease occurred in 7 breeding sheep in the eastern region of Morocco. Thirty three local sheep breed were clinically infected and five of them died, of which four were lambs of two months old. These animals were transported to the laboratory for autopsy and organs collection was performed in good conditions.

For the molecular diagnosis, the clinical samples such as nasal, ocular and rectal swabs (33 samples of each), blood (28 samples) and crusted scabs (4 samples) were collected and transferred to the laboratory under chilled conditions without transport medium. During the necropsy, five lung nodules samples were taken for virological investigation and molecular characterization. Race, age and sex were not considered.

Ocular swabs, lung tissues and mediastinal lymph nodes were also tested for the investigation of peste des petits ruminants (PPR), but all specimens were negative for this disease.

### Virus isolation

Isolation of SPPV in lung nodules samples was taken from autopsied lamb and performed on lamb testis cells (TA) as described in World Organization for Animal Health (OIE) protocol [10]. An amount of 200 μl of infectious filtrate was used to infect TA cells in 24-well plates in duplicate with negative controls. The infectious filtrate was obtained by lung nodule mincing in a sterile sand with meduim MEM using a mortar followed by filtration of the solution in 0.45 mm. Cells were examined for cytopathic effect (CPE) for 14 days after infection. The contents of negative wells were collected and freezed-thawed. The supernatant was sonicated and centrifuged. Samples were then used to infect fresh TA cells, which were examined for another 14 days postinfection of CPE.

### DNA extraction

The swabs were swirled using 2 ml of PBS 10%. After spinning, the obtained liquid was transferred to a fresh sterile tube. Crusted scabs and lung nodules were minced using a pestle and 4 ml of 10% sterile PBS. The mixtures obtained were then centrifuged for 10 min at 2000 rpm. The supernatant solutions from this step were collected and kept frozen at -20°C until DNA extraction. Steps were carried out within in a Microbiological Safety Cabinet IIA. The blood and purified vaccine virus have been used directly for DNA extraction. The extraction of the viral DNA was performed by the QIAamp DNA Mini Kit QIAGEN according to the manufacturer’s instructions.

### Real-time PCR assay

The PCR method developed by Balinsky *et al.*[12] was used. Briefly, reagents from an EZ-RT PCR kit (Applied Biosystems), a forward primer (5′GGCGATGTCCATTCCCTG 3′), reverse primer (5′AGCATTTCATTTCCGTGAGGA3′) and TaqMan probe (5′CAATGGGTAAAAGATTTCTA3′) labeled at the 5′end with reporter dyes FAM were used in the following cycling conditions: initial denaturation at 95°C for 120 seconds, followed by 45 amplification cycles (95°C for 2 s and 60°C for 60 s). The assay was run on a Cepheid SmartCycler (Cepheid, Inc., Sunnyvale, CA).

The primers and probes were synthesized by Operon Biotechnologies Gmbh, Germany. All clinical samples were retested by conventional PCR using InS-1.1 left and InS-1.1′right primers [13], as previously described by Mangana-Vougiouka *et al.* and Zro *et al.*[14,15].

### Molecular characterisation (PCR and phylogenetic study)

In this section, the isolated SPPV strain from lung nodule was identified by PCR as described by Balinsky [12] as well as the vaccine strain used in Morocco (Romania 65).

The targeted genes for sequencing were: the TK gene encoding thymidine kinase [13] and the portion 3 L of chemokine analogue receptor of interleukin gene (IL8) [16]. The used primers for the TK gene were (TK7, TK8), (TK11, TK12) and (Chim1, Chim2) were used for the portion 3 L of interleukin 8 gene, flanking regions of 680, 420 bp and 1200 bp, respectively [17].

The PCR was carried out in a volume of 25 μl containing 10 μM forward primer, 10 μM reverse primer and 1 μl viral DNA using Taq platinium Invitrogen kit. The thermal cycler protocol was at 96°C for 4 min followed by 35 cycles of 96°C for 10 sec; 50°C for 40 sec and 72°C for 2 min and one final extension step at and 72°C for 4 min. Thermal cycler “Verity” of ABI was used for PCR reactions. The PCR products were submitted to electrophoresis on a 1% agarose gel. The amplified products were visualized by the system of photo-documentation “G Box”.

PCR products were purified by using ExoSAP-IT treatment (USB Corporation, Cleveland, OH, USA), and were used as template for direct sequencing. Bi-directional sequencing was performed by using a BigDye terminator cycle sequencing kit (Applied Biosystems, Foster City, CA, USA) and an ABI PRISM 3130XL automated DNA sequencer.

Pure DNA was used at a concentration of 100 ng/μl for each sample and each reaction in a final volume of 10 μl. The thermal cycler protocol was: initial denaturation at 96°C for 1 minute and 10 seconds of denaturation at 96°C, 5 seconds annealing at 50°C, 4 minutes of elongation at 60°C for 25 cycles.

### Phylogeny analysis

Ten viral strains of CaPV were used in this study. Among them the Moroccan field strain, Moroccan vaccine strain and eight were publically available in GenBank database and were accessed in December 2012. The construction of two phylogenitic trees was performed using both of TK and IL8.

Alignment was done with ClustalX [18]. A phylogenetic tree was made using Bootstrap neighbour-joining method and visualized by NJPlot.

### Ethical approval

This work is done in the context of regulatory controls prescribed by ONSSA. The ONSSA (National Office of Food Safety products) is the national body responsible for controlling plant and animal diseases, products derived from plants and animals, foodstuffs intended for animal feed, veterinary drugs or any other product intended for use medicine and veterinary surgery. In this work, sampling and autopsies are performed on animals, according to the guideline of the ONSSA.

## Results and discussion

### Sheeppox outbreaks

The SPPV is an infectious transboundary disease that negatively impacts on animal trade and causes significant economic losses, so it must be notified to the OIE [3].

In Morocco, sheeppox is endemic despite the implementation of a control program based on sanitary prophylaxis associated with vaccination by live attenuated vaccines locally produced [19]. However, during 2010, the epidemiological rate changed due to the emergence of SPPV epizootics outbreaks in different regions of the kingdom.

Despite the presence of goats in the same flock as affected sheep, in all outbreaks, the clinical signs were observed only in sheep. This assumes that the SPPV Moroccan wild strain has a strictly a sheep tropism which is contrary to what has been recently reported in China where GTPV was isolated from sheep [6]. The average Morbidity (based on clinical signs) was 4% and average mortality was 1.25% and was essentially observed in lambs (4 cases/5). The common clinical form of sheeppox disease observed in Morocco is the classical vesicular form [5]. However, in this study, we reported for the first time in Morocco a new clinical form called the nodular form. The clinical signs in sheep included mild to severe clinical diseases. Tested animals were suffering from fever, depression of appetite, serous to mucopurulent nasal discharge in adult subject and the evolution of clinical signs was fatal in lambs.

**Figure 1:**
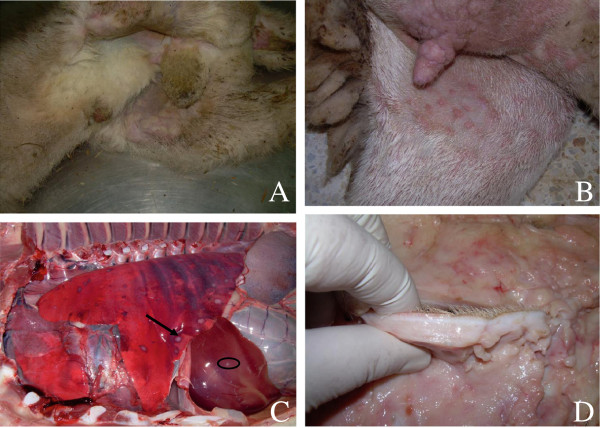
**Characteristic symptoms of the new clinical form of sheep pox disease in Morocco. (A)** Typical nodules lesion in internal face of the thigh of the lamb; **(B)** nodule lesions like tumor skin on the teat and udder affected with sheep pox virus; **(C)** Typical pox lesions observed on the lungs and liver of a lamb; **(D)** Acute inflammatory reaction associated with hyperplasia of the skin caused by wild strain SPPV.

On the lesion, we noted the development of erythematous macules, papules and nodules (varying in size from 0.5 to 2 cm) on the skin, udder, vulva, testicles, foreskin, the inner side of the thigh and in the whole free part of the skin. These nodules could confluence and form lesions that look like skin tumors (Figure 1A & B), the nodular lesions were increased in number and when palpated felt hard. No vesicular changes were observed during the progression of the lesions. Moreover, the surviving animals showed skin lesions and scabs toward the end of disease evolution.

Interestingly, the nodular form has already been reported in some countries, especially in West Africa, including Mauritania [20] and other parts of the world, where the disease is endemic [2].

At necropsy examination, internal lesions as nodules were found in the lungs in all cases, consistently in the liver, kidney and gastrointestinal mucosa (Figure 1C), causing different signs like respiratory signs and diarrhea, specially observed in lambs. Dramatic inflammatory reaction in the skin was also observed (Figure 1D), this perhaps was related to strong epithelial tropism of field Moroccan SPPV strain.

Indeed, it has been reported in Turkey a natural infection with a strain of SPPV causes a hyperplasia with hyperkeratosis of skin and acanthosis of the stratum corneum. In addition, the dermis showed epithelial hypertrophy and nuclear pyknosis in wool follicles, sebaceous and sweat gland [9].

### PCR analysis

Control and eradication of sheeppox disease depend on accurate diagnosis and vaccination in endemic countries. Therefore, a rapid, sensitive diagnostic tool for screening affected sheep flocks is essential [21]. The traditional diagnosis of SPPV is based on clinical manifestations, virus isolation, electronical microscopy and serology. The clinical manifestations require a laboratory confirmation, the virus isolation is time consuming, Electronic microscopy is not a method for routine purposes, and serological assays lack sensitivity and specificity [2,22]. For all these inconveniences, an alternative approach is mandatory.

In the last decades, molecular techniques have been well established for the diagnosis of infectious diseases. Among molecular techniques, PCR, which has been successfully used for the diagnosis of SPPV diseases and considered one of the best alternatives of conventional assays due to its sensitivity, specificity and reproducibility [14,23].

Conventional PCR assays have successfully been employed for routine diagnosis using different types of samples [15], but with additional time for manipulation of post-PCR products on agarose gel and high risks of cross contamination when the number of tested samples is high. Real-time PCR assay is more rapid, specific and sensitive than traditional PCR and has been developed in several laboratories [12,24,25].

The nodular form of sheeppox disease outbreaks in Morocco provided an opportunity to evaluate the use of conventional types of samples such as nasal, ocular, rectal swabs, blood, skin scabs and lung nodules for molecular diagnosis by using quantitative PCR. In the past, the diagnosis of SPPV has traditionally been based on clinical diagnosis and serological assays such as VNT, which demonstrate the presence of specific SPPV antibodies in the serum of animals, even with vaccination. However, the emergence of other diseases such as PPR requires the confirmation of the suspicion by the use of molecular tools.

Among the objectives of this work was to evaluate the clinical sensitivity and specificity of the PCR developed by Balinsky *et al*[12] in animals naturally infected with the nodular form of sheep and healthy animals using nasal swabs, ocular swabs, rectal swabs, blood and crusts scabs. The clinical sensitivity of the test using crusts was 100%, this type of sampling can be considered as the best clinical specimen for diagnosis of SPPV. However, the presence of this type of lesion was observed in infected animal in the terminal stage of the disease, while young animals succumb during the phase of the development of skin nodules.

Effective management requires a rapid confirmation of outbreaks infection of sheep. For this purpose, nasal swabs, ocular swabs, rectal swabs and blood were evaluated (Table 1). The clinical sensitivity for the real-time PCR assay on ocular swabs, nasal swabs, rectal swabs were 96.97 (32/33), 93.94 (31/33), 90.91 (30/33) respectively, with a specificity of 100% (Table 2). However, in blood samples, the sensitivity was 66.67 (Table 2) and blood can not be considered a good sample for PCR diagnosis of SPPV, likely due to the low viremia observed in SPPV infection. We also confirmed that the PCR was able to detect virus in the lung modules obtained from animals succumbed of the disease.

**Table 1:** Results (threshold cycle values) of real-time PCR obtained from suspected clinical samples

**Identification number**	**Nasal swab**	**Ocular swab**	**Rectal swab**	**Blood**	**Crusted scab**	**Lung nodule**
	**TaqMan assay**	**TaqMan assay**	**TaqMan assay**	**TaqMan assay**	**TaqMan assay**	**TaqMan assay**
	**(Ct value)**	**(Ct value)**	**(Ct value)**	**(Ct value)**	**(Ct value)**	**(Ct value)**
1 (outbreak n° 1)	25,93	25,97	30,83	36,2	19,88	-
2 (outbreak n° 1)	27,57	27,8	32,77	38	22,88	-
3 (outbreak n° 1)	29,12	31,08	28,32	37,6	-	-
4 (outbreak n° 1)	30,28	30,83	40,03	30,75	-	-
5 (outbreak n° 1)	31,97	32,8	35	39,87	19,59	-
6 (outbreak n° 1)	38,33	26,07	38,93	37,78	-	-
7 (outbreak n° 1)	25,82	39,75	33,6	33,42	-	-
8 (outbreak n° 1)	No Ct	42,5	No Ct	-	-	18,7
9 (outbreak n° 1)	39,93	No Ct	39,21	37,1	-	-
10 (outbreak n° 1)	38,3	22,97	30,32	37,8	-	-
11 (outbreak n° 1)	24,03	24,5	37,17	40,93	20,98	-
12 (outbreak n° 1)	21,56	35,95	No Ct	-	-	17,95
13 (outbreak n° 2)	35,07	33,25	38,78	41,1	-	-
14 (outbreak n° 2)	29,8	28,83	No Ct	36,5	-	-
15 (outbreak n° 2)	30,69	34,64	42,5	30,29	-	-
16 (outbreak n° 3)	36,64	31,21	35,6	No Ct	-	-
17 (outbreak n° 3)	34	29,86	36,04	No Ct	-	-
18 (outbreak n° 3)	29,7	33,5	38,75	-	-	19,93
19 (outbreak n° 3)	34,2	33,93	34,93	40,75	-	-
20 (outbreak n° 3)	33,92	36,33	38,2	36,4	-	
21 (outbreak n° 4)	35,07	37,9	39,5	33,96	-	
22 (outbreak n° 4)	40,48	40,16	41,1	-	-	20,37
23 (outbreak n° 4)	41,7	35,87	35,1	No Ct	-	-
24 (outbreak n° 4)	40,16	35,83	34,83	38,04	-	-
25 (outbreak n° 4)	35,66	33,3	39,5	35,86	-	-
26 (outbreak n° 5)	No Ct	33,95	35,32	No Ct	-	-
27 (outbreak n° 5)	37,5	31,39	40,3	40,68	-	-
28 (outbreak n° 5)	33,96	30,42	39,5	-	-	22,94
29 (outbreak n° 6)	27,5	27,5	38	No Ct	-	-
30 (outbreak n° 6)	40	27,18	39,5	40,67	-	-
31 (outbreak n° 6)	24,58	22,28	38	No Ct	-	-
32 (outbreak n° 6)	19,39	26,8	29,89	39,48	-	-
33 (outbreak n° 7)	28,79	22	26,62	33,84	-	-

-° : sampling unrealized.

**Table 2:** The sensitivity of the PCR on different clinical samples

**Type of sample**	**Ocular swab**	**Nasal swab**	**Rectal swab**	**Blood**	**Skin crust**	**Lung nodule**
Sensitivity of PCR (%)	96,97 (32/33)	93,94 (31/33)	90,91 (30/33)	66,67 (22/28)	100 (4/4)	100 (5/5)

The results of testing 136 clinical samples by conventional PCR, and real time PCR reveal that 88 (64.7%) and 124 (91.2%) were positive in conventional and real time PCR assays, respectively. These results indicate that both assays are adequate for the detection of SPPV from skin scab, lung nodule, nasal swab, ocular swab, rectal swab samples. The results also showed high diagnostic efficacy of real time PCR than conventional PCR (data not showed).

The real time PCR previously described by Balinsky [12] is a potential tool and could be used as a replacement or complementary test of conventional assays for the diagnosis of the nodular form of sheeppox disease in Morocco.

### Molecular characterization

In order to fight against SPPV, the veterinary health authority in Morocco practices regular vaccination with live attenuated Romania strain 65 vaccine. The recorded outbreaks of sheeppox disease in the first half of the year 2010 as epizootics peaks, especially with the emergence of unusual clinical nodular form has led us to associate a PCR based automated sequencing study on the part of the genomes of the Romania 65 vaccine strain and the new isolated SPPV strain.

**Figure 2:**
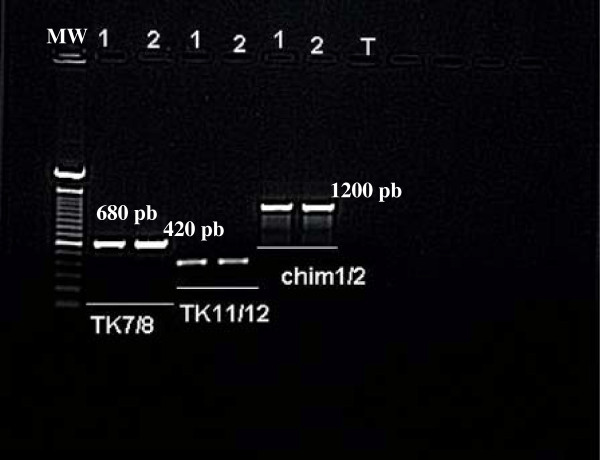
**Specific amplification of the TK gene by two pairs of primers and receptor gene analogous to IL8 by a single primer pair.** 1: vaccine Romania 65 strain. 2: isolate field strain. MW: Molecular Wight.

Genomic PCR identification of both strains was performed targeting TK and IL8 genes. The specificity and sensitivity of PCR was widely proved by Ireland and Binepal and Fakhfakh et al for CaPV identification [17,23]. The IL8 is a member of the subfamily of C-X-C chemokines; it is involved in the immune response by stimulating the activity of neutrophils [26], while the TK gene is essential for poxvirus multiplication [27]. The use of two pairs of primers (TK7, TK8) and (TK11, TK12) showed an amplification of 680 bp and 420 bp respectively, whereas the detection of the analogous receptor IL8 gene was performed by using a single primer pair (Chim1, Chim2), which showed an amplification of 1200 bp in both strains (Figure 2). The nucleotide sequences obtained are registered in the Genbank database under the following accession numbers: KC756805; KC756806; KC756807; KC756808 correspond to IL8 gene of wild and vaccine strains and TK gene of wild and vaccine strains respectively.

Sequencing of targeted genes showed that the length of the sequenced DNAs obtained was similar for both of vaccine Romania 65 strain and isolated SPPV strain. It also helped to highlight one single base substitution (A/T) at the TK gene. The conversion of sequences to amino acid showed that it is a silent mutation (M/Y).

**Figure 3:**
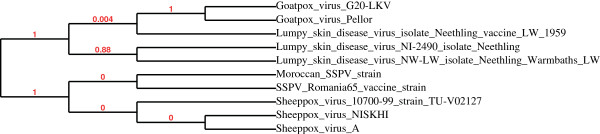
**Phylogenetic tree of TK.** Strains of SPPV species are shown in a separated outlier and strain of interest is clustering with the vaccine Romani 65 strain (Moroccan vaccine).

**Figure 4:**
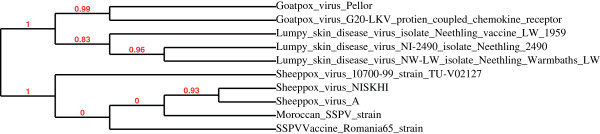
**Phylogenetic tree of receptor gene analogous to IL8.** Strains of SPPV species are clustering together, while Romania 65 is presented by a separated clade.

Phylogenetic trees based on the TK and the portion 3 L of chemokine analogue receptor of interleukin IL8 sequences are shown in Figure 3 and Figure 4. In both constructed trees, genomes belonging to the SPPV clearly cluster together; while strains of other CaPVs are displayed by a separated outlier and this obviously shows the high degree of similarity and genetic relationship among SPPV strains and this could prove that these viruses descended from the same ancestor. Significantly, strains of interest occurs in a cluster with other SPPV by using the sequences of portion 3 L of analogous IL8 receptor and the vaccine strain was completely displayed by a separated clade, one other hand the strain of question has been clustered with the vaccine strain, when TK sequences were treated for the construction of the phylogenetic TK gene sequences based tree for the same viral strains.

This clearly shows the importance of including other genes and extends this study with more SPPV species for building an accurate visualization of their phylogeny.

Our results showed a sequence conservation between the vaccine and SPPV strains, despite the spatial and temporal diversity. At the same time, it would be useful to carry out more detailed analysis of other gene fragments and future strains isolated in different regions of Morocco to help explain the change of the clinical expression of sheeppox viruses.

## Conclusion

This work has elucidated the nodular form of sheeppox reported for the first time in Morocco and identified the wild strain SPPV responsible for the clinical expression of this form. We have also demonstrated that the real-time PCR on clinical samples from experimentally infected animals can be successfully used on samples from naturally infected animals using different types of samples.

In addition, the detection of thymidine kinase gene (TK) and chemokine analogue receptor of interleukin gene (IL8) was observed in the field and the vaccine strains and their genetic profile were highly identical. These results have highlighted the conservation of these genes and thus has leaded to further study and comparison among other genome sequences of these strains. This finding may provide a new insight on the epidemiology of sheeppox disease in Morocco.

## Competing interests

The authors declare that they have no competing interests.

## Authors’ contributions

KZ performed, analyzed the data and wrote the first draft of the manuscript. FZ analyzed the data and participated in the design of the study and data interpretation. MM and EEF made substantial contribution to conception and design of the study. MME conceived of the study and helped drafting the manuscript. All authors read and approved the final manuscript.
